# Notes and News

**Published:** 1889-01-12

**Authors:** 


### NOTES AND NEWS.

COLLECTED AND COMMUNICATED.

A NEW idea for helping hospitals was carried out by the West London Carol Singers. This band, consisting of 20 volunteers, both ladies and gentlemen, went singing for five nights during the fortnight before Christmas in the streets of Kensington between the hours of seven and eleven, and the result is that they were able to add the handsome sum of £100 to the funds of the West London Hospital. Other districts possessed of musical talent, please copy this example next year.

AN interesting feature of the Christmas fête at the German Hospital, Dalston, was a portable fountain which played a jet twenty-one inches high, and was the work of one of the patients. He is a coppersmith by trade, and some time ago fell into a large vat he was repairing, and sustained rather severe injuries. Since he has been more or less convalescent, he has been turning his skill to account for the benefit of his fellow-patients. Besides the fountain which has been mentioned, he has made windmills and other clockwork toys for the children's ward.

IT is proposed that the council of Owens College and the committee of the Manchester Royal Infirmary should hold a conference with regard to the proposed new hospital of the Whitworth legatees. The council would like to have a hospital somewhere nearer it than the infirmary in Piccadilly for convenience of instruction to the students, while, on the other hand, they do not wish to do anything to injure the existing institution. It is to be hoped that a feasible *locus standi* will be arrived at, and that a gift valued at £70,000 may be made available both for the advancement of medical science and the aid of the sick poor of the capital of Cottonland.

THE Christmas entertainment to the small patients at the Paddington Green Children's Hospital took place, to state it in Hibernian fashion, in the New Year—to wit, on the 3rd inst. It was of a varied character. Mr. M. Beachcroft gave a magic lantern entertainment, with an illustrative reading. In the largest ward there was a performance of some obedient birds, supported by very talented and amicable cats and white mice. Without going into the performance in detail, it may be said, to use Mr. Anstey's words, that "them tabbies sparred with a science you'd hardly expect in sich; and the mouse, what usually boggles, fetched flags and never no hitch"—even when the flagstaff was held in the somewhat unsteady hand of invalid children. The matron, Miss Anderson, was able to be present, though the fatigue of receiving so many visitors and looking after her excited patients must have tried her strength not a little. There was only one thing to be regretted—that in spite of the recent enlargement of the out-patient department of the hospital, it is still so small. Paddington is large enough to require a bigger children's hospital; Paddington is rich enough to support it.

THERE has been some correspondence in the *Times* on flowergirls and their trade, one writer ("C.F.R.") being anxious to persuade the public not to buy flowers in the street, because when their wares are not all sold in the street the women—it is absurd to talk of them as if all were young girls—try to dispose of the remainder in public houses, whence evils of various kinds may arise. The logic of the appeal is not very clear. If none of the flowers are bought in the street, the public-houses will be resorted to the more; or if "C.F.R." gets his own way and the women are starved out of selling flowers in the street, what other honest trade are they fit for? It is very risky to stop any industry, honest in itself, because of the possible moral dangers that underlie it. Is there any occupation where temptation is unknown?

Is it justifiable, or even decorous, in Mr. George Woodcock to write to the *Coventry Mercury*, asking the subscribers to the Coventry and Warwickshire Hospital to elect Dr. Phillips to the vacant post of honorary medical officer to that institution, on the plea that he is the nephew of his uncle? The late Dr. Phillips was doubtless a skilled and generous man, but that is no reason for selecting his nephew in preference to other equally-suitable candidates. The present Dr. Phillips, we are assured, has testimonials of the highest class, and is altogether worthy of the position he seeks, but if so he will be all the more annoyed at the conduct of his well-meaning but injudicious friend Mr. Woodcock, who, without consulting him, has taken a step much more likely to mar than to aid his candidature.

ON Thursday, December 27th, thanks to the beneficence of kind friends, Miss Wagnell, matron of the Congleton Cottage Hospital, was enabled to provide for her patients, as well as for a large number of former patients and children, a sumptuous Christmas tea. It is customary to speak of well-spread tables as "groaning" under their weight of good things; but if we are to endow tables with feeling, we would rather say they rejoiced at being the bearers of the good cheer provided for these sick and poor folk. Ham sandwiches, pork pies, plum cake, mince pies, buns, and a variety of other dainties tempted and gratified the palates of Miss Wagnell's happy guests. The tables were presided over by the Mayoress (Mrs. Moss), Mrs. Besant, Miss Bannerman, Mrs. Conder, Mrs. Davidson, Mrs. Farrington, Mrs. Fielder Hall, Mrs. Phelps, Mrs. H. L. Reade, the Misses Reade, Miss Twells, and Miss Washington. After tea the company adjourned to the gallery and wards of the hospital, which were tastefully decorated for the occasion, and here music and recitation were well and cheerfully given to the delighted guests. The Mayor (Dr. Moss), who is one of the hospital doctors, delighted all ears with his skilful production of silvery notes from what looked like a silver flute. To the uninitiated his playing seemed marvellous. The Mayoress, too, not only presided at the piano, but gave several songs in good taste, and other friends helped to make this informal concert a success. Then came the distribution of gifts from the bran tub. The pleasant task of distributing these Christmas gifts was, with businesslike despatch, performed by Dr. Moss and Mr. C. R. Hall. At this part of the entertainment most of the clergymen of the neighbourhood were present. At the conclusion of the proceedings the Rev. W. W. Thackray, on behalf of the matron, patients, and visitors, thanked Dr. Moss and those who had been associated with him for their kindness in providing so excellent an evening's entertainment. On leaving, each person who during the year had been an inmate of this hospital was presented with warm articles of clothing and bags containing fruit and confectionery. If looks mean anything, the matron, donors, and workers who provided this treat were most eloquently thanked by the recipients of their kind gifts, who looked gratitude itself.

The following vacancies are announced this week, the amount of salary in each case being given after the name of the institution :—

**Resident Medical Officers.**

Chelsea Hospital for Women, Fulham Road.—£80 per annum, with Board, etc.
City of London Hospital for Diseases of the Chest, Victoria Park, N.E. Resident Clinical Assistant.
County Clare Infirmary. Surgeon.—£94 per annum.
Durham County Asylum. Second Assistant Medical Officer.—£150 per annum, with board, etc.
East London Hospital for Children, Shadwell, E. Resident Clinical Assistants.—Board, no salary.
General Hospital for Sick Children, Pendlebury, Manchester. Junior Resident Medical Officer.—£80 per annum, with board, etc.
Hants County Asylum. Junior Assistant Medical Officer.—£100 per annum, with board, etc.
Nottingham General Dispensary. Senior Resident Surgeon.—£180 per annum.
Radcliffe Infirmary, Oxford. House Surgeon.—£80 per annum, with board, etc.
Reading Amalgamated Friendly Societies Medical Association. Resident Medical Officer.—£200 per annum, with fees.
Royal Hospital for Diseases of the Chest, City Road, London. House Physician—£50 per annum, with board.

**Non-resident Medical Officers.**

Lodge of Oddfellows of the Manchester Unity. Medical Officer.
Friendly Societies of Eyam, Sheffield. Qualified Medical man.—£55 per annum.
Leicester and Rutland. Medical Officer of Health.—£450 per annum.
Manchester Royal Infirmary. Honorary Assistant Physician.
Victoria Hospital for Sick Children, Queen's Road, Chelsea, S.W. Physician to in patients.

---

The annual Christmas festivities in connexion with the North Staffordshire Infirmary comprised a musical and dramatic entertainment, given on December 26th, to the patients and hospital staff, in which the three resident medical officers took part. A dance on January 2nd was also included, at which about one hundred of the friends of the nurses were present. Both the entertainments were held in the large out-patients' room, the dimensions of which are 95 ft. by 35 ft. The profuse decorations were displayed to perfection. The music was supplied by a band of four performers. The cost of the entertainment was defrayed by private subscriptions, collected by the matron, Miss Annan, to whom great praise is due.

---

A very successful entertainment was held on Saturday, December 29th, at the Samaritan Free Hospital. The proceedings were commenced by a sumptuous tea, provided in the board-room for the nurses and old patients. The large ward in which the entertainment took place was filled by a number of old and present patients, several members of the medical staff, and numerous ladies and gentlemen who take an interest in the hospital, or who helped to carry out the programme. The matron, Miss Butler, is to be congratulated upon the amount of talent which she was able to draw together to amuse and entertain her visitors and patients, whose applause gave sufficient evidence of their enjoyment. It would be invidious to select any of the performers for special mention, but we cannot refrain from noticing with pleasure that the charming actress, Mrs. Laurance D'Orsay (herself once a patient at the hospital) spared time from her professional duties to assist her husband, Mr. Laurance D'Orsay, in a bright comedietta, entitled "The Happy Pair." In the absence of another performer, Mr. Sidney Herberte-Basing, from the Shaftesbury Theatre, kindly gave a brilliant rendering of a new and expressive song, "My Friend." An amusing item of the entertainment was the distribution of a number of useful and ornamental presents by Father Christmas (Mr. A. O. Collard) gorgeously attired and decorated in conventional style, who was assisted in his duties by little Fairy New Year (Miss Constance Bantock) who won all hearts by her appearance, and confirmed her victory by the gifts she was the medium of presenting.

A few days ago a patient in Castleblaney Fever Hospital, when delirious from typhus fever, jumped through a ward window, carrying part of the frame with him in his exit. He was captured in a neighbouring field, apparently none the worse for his airing.

---

Liverpool is proposing to have a Nurses' Pension Fund of its own. The idea was suggested some time ago, but it was found impossible to obtain a fund large enough to pension the nurses, even though it was meant to conduct it on provident principles. To prevent so good a thing failing, however, Mr. James Harrison has now come forward and promised £5,000 if the fund can be placed on a sound footing, while on the same conditions Mr. Tate and Mr. Rathbone have promised £1,000 each. It is well to look after one's own, but would not Messrs. Harrison, Tate, and Rathbone be doing better if they put their generous contributions into the National Pension Fund, the success of which is now assured? Other towns are doing so, and the constitution of the fund permits that Liverpool contributions should be applied to the pensioning of Liverpool nurses by Liverpool trustees, while the large-hearted millionaires of the great Lancashire seaport would be saved the anxiety, worry, and possible failure of a local fund.

---

A fortnight ago the *Lancet* made some very severe remarks on the number of beds in the new Birmingham workhouse infirmary, as too large to admit of proper attention being paid to the patients by the medical staff, which it said numbered only four gentlemen. To the *Lancet* criticisms the visiting physician to the infirmary, Dr. Suckling, has replied that though the infirmary will ultimately contain 1,700 beds, for some time to come 1,400 will be the maximum number available; that at present 300 of the patients are epileptics, able to go about and be at work; that five-sixths of the cases admitted are chronic and do not need daily examination; and finally, that the medical staff numbers six individuals, not four. Dr. Suckling also invites the *Lancet* to send a representative to investigate the medical administration of the infirmary and report on it; but the explanations he has already given will probably convince the author of the criticisms in question that it is well to look before you leap into faultfinding with an institution with which you are not intimately acquainted.

---

A correspondent writes: At Christmas the Poplar Hospital had its week of gladness and rejoicing. The members of the choir of Allhallows, who had kindly arranged to come to the hospital on Christmas morning to brighten for an hour the poor sufferers, were arranged on the stairs, so that all the patients had the benefit of hearing the singing. The carols were followed by a seasonable dinner of roast beef and plum pudding. Each male patient received a present of tobacco and a pipe, and had the privilege of smoking to his heart's content; and all were present at a concert in the evening. On Boxing Day, in Buxton (a children's ward), there was a large Christmas tree loaded with presents for young and old. To see the bright and happy faces of the poor little invalids, one could hardly believe they were suffering from serious accidents, or were the sick inmates of a hospital. Each had a suitable present and a pretty toy along with it. The adults received gifts of good warm clothing, and the matron had thoughtfully prepared something for each of the children of the patients who came to see the sights and cheer their relatives. After the tree there was a concert in Gurney Ward, the entertainments being concluded on Thursday, when the clergy of Allhallows gave a magic lantern entertainment, which was very much enjoyed. Great praise is due to the matron, the house surgeons past and present, and also to other friends, who were untiring in their efforts to make all the patients happy and forgetful of suffering for a brief period at any rate.

